# Methotrexate-Induced Optic Neuropathy: A Rare and Under-Recognized Cause of Visual Loss in the Elderly

**DOI:** 10.7759/cureus.97836

**Published:** 2025-11-26

**Authors:** Mubshra Tariq, Rutbah Amin Khairati, Narender Singh, Syeda Nafisa

**Affiliations:** 1 Acute/Internal Medicine, George Eliot Hospital NHS Trust, Nuneaton, GBR; 2 Internal Medicine, George Eliot Hospital NHS Trust, Nuneaton, GBR; 3 Rheumatology, George Eliot Hospital NHS Trust, Nuneaton, GBR; 4 Respiratory Medicine, Nottingham University Hospitals, Nottingham, GBR

**Keywords:** drug-induced neurotoxicity, drug-induced optic neuropathy, elderly patient care, methotrexate neurotoxicity, psoriatic arthritis, vision loss

## Abstract

Methotrexate (MTX) is widely used in the management of psoriatic arthritis and is generally considered a safe disease-modifying antirheumatic drug (DMARD). Although its systemic adverse effects are well established, neurotoxicity, including optic neuropathy, is exceedingly rare and often overlooked. This report describes the case of a 74-year-old man on long-term low-dose MTX who presented with progressive unilateral visual loss. Examination revealed optic disc pallor, retinal nerve fiber layer (RNFL) thinning, central scotoma, low serum folate levels, and borderline vitamin B12. Magnetic resonance imaging (MRI) excluded demyelinating or structural pathology. MTX was discontinued, and vitamin supplementation was given, stabilizing vision but with persistent optic atrophy. This case underscores the importance of clinician awareness and early ophthalmic evaluation in MTX-treated patients presenting with visual symptoms, especially those in high-risk groups.

## Introduction

Methotrexate (MTX) functions as a first-line disease-modifying antirheumatic drug (DMARD) used for rheumatoid arthritis, psoriatic arthritis, and various dermatologic conditions. Although generally well tolerated when administered with folic acid supplementation, MTX is associated with several adverse effects, including hepatotoxicity, bone marrow suppression, pneumonitis, and neurotoxicity [1].

MTX exerts its pharmacologic effect by inhibiting dihydrofolate reductase and impairing the conversion of dihydrofolate to tetrahydrofolate, an essential cofactor in the synthesis of purines, thymidylate, and methyl donors such as S-adenosylmethionine. The disruption of these pathways leads to impaired DNA repair, reduced myelin synthesis, and altered neuronal methylation [2,3]. Additionally, MTX interferes with mitochondrial oxidative phosphorylation, compromising axonal metabolism, which is particularly consequential for the highly energy-dependent optic nerve [4]. Elevated homocysteine levels, common in folate and vitamin B12 deficiency, may further exacerbate toxicity by inducing oxidative stress, endothelial dysfunction, and microvascular ischemia [5,6].

While MTX-associated neurotoxicity more commonly manifests as leukoencephalopathy, seizures, or stroke-like episodes, optic neuropathy remains an exceptionally rare presentation. A 2020 systematic review found ophthalmic toxicity from systemic MTX so uncommon that no reliable incidence could be established [7,8]. Nonetheless, published case reports highlight that elderly individuals on long-term low-dose MTX may be particularly vulnerable, especially when additional risk factors such as nutritional deficiencies, gastrointestinal malabsorption, chronic alcohol use, or genetic polymorphisms in folate metabolism (e.g., methylenetetrahydrofolate reductase {MTHFR} variants) are present [9-11].

Given that optic neuropathy may present insidiously, with unilateral or bilateral blurred vision, dyschromatopsia, or visual field defects, early recognition is crucial. Delay in diagnosis can result in irreversible optic atrophy; however, the timely discontinuation of MTX and aggressive folate/B12 supplementation have been shown to halt progression and, in some cases, reverse visual impairment [9,10]. Therefore, clinicians must maintain a high index of suspicion for visual symptoms in patients receiving long-term MTX therapy, particularly within high-risk groups.

## Case presentation

A 74-year-old man with known psoriatic arthritis, taking methotrexate 15 mg weekly and folic acid 5 mg weekly for five years and two months (total intake: 3930.0 mg), presented with progressive visual blurring in the right eye over the course of three months. The patient's medical history was characterized by ischemic heart disease, as well as hypertension. The patient has had no fresh infection, injury, surgical operation, or any other signs of an underlying disease before the beginning of the indexed course. On the ophthalmological examination, visual acuity (uncorrected) in the right eye was reduced to 6/36 and in the left eye 6/12, with no relative afferent pupillary defect (RAPD) in either eye and normal extraocular movement (EOM). The right optic disc was pale, and there was no sign of papilledema or hemorrhage in the right and left eyes. Intraocular pressure in the right eye was noted to be 18 mmHg and in the left eye 19 mmHg, both within normal range. Automated perimetry demonstrated a peripheral visual field defect affecting the inferior, nasal, and superior quadrants of the right eye, with a normal field in the left eye. The assessment of color perception using Ishihara plates revealed normal color vision bilaterally.

**Figure 1:**
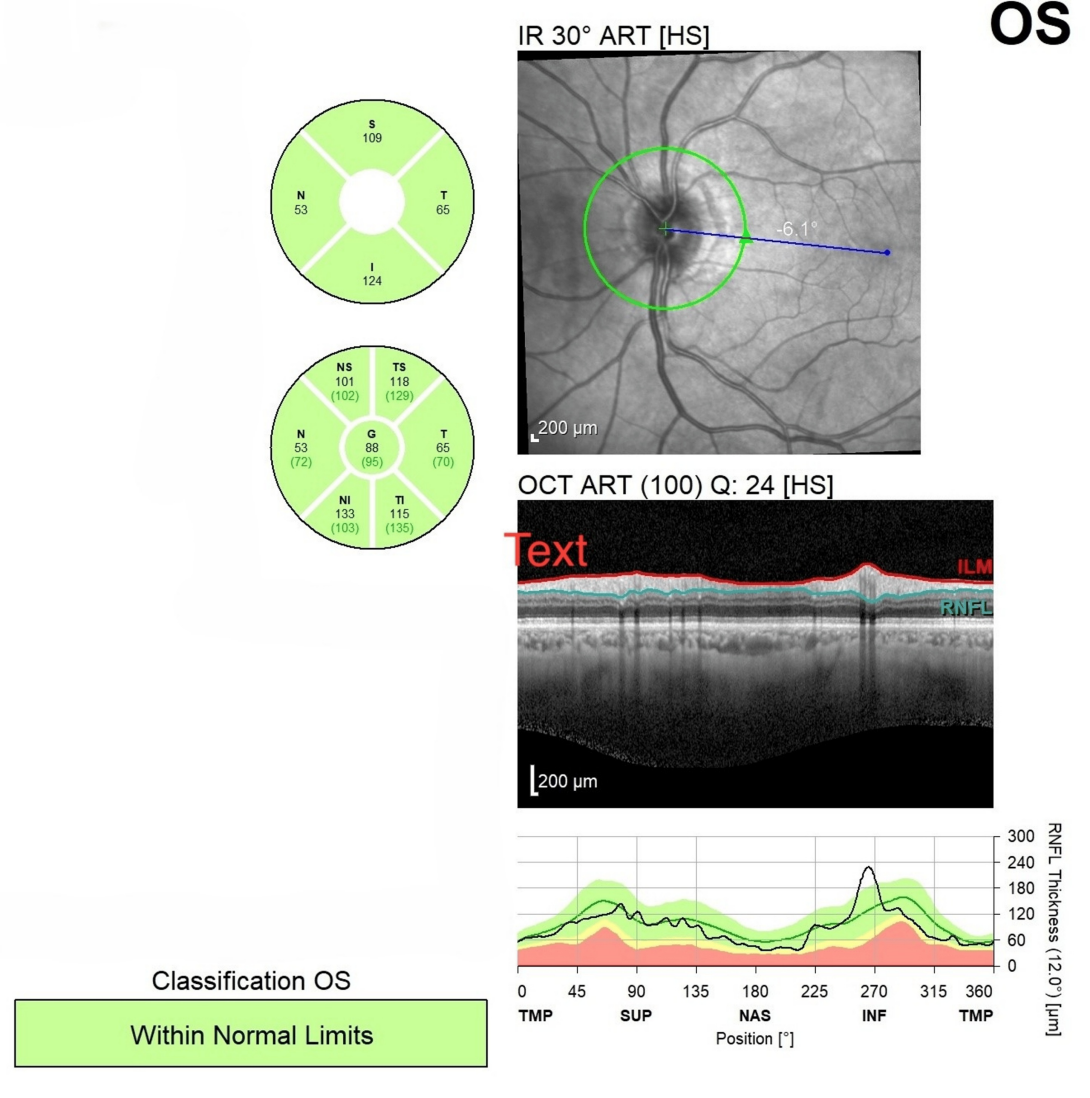
Optical Coherence Tomography (OCT) of the Left Eye (OS) Manifesting Normal Retinal Nerve Fiber Layer (RNFL) Thickness ART, automatic real time; HS, high sensitivity; S, superior; N, nasal; T, temporal; I, inferior; G, global; TMP, temporal-macular profile

**Figure 2:**
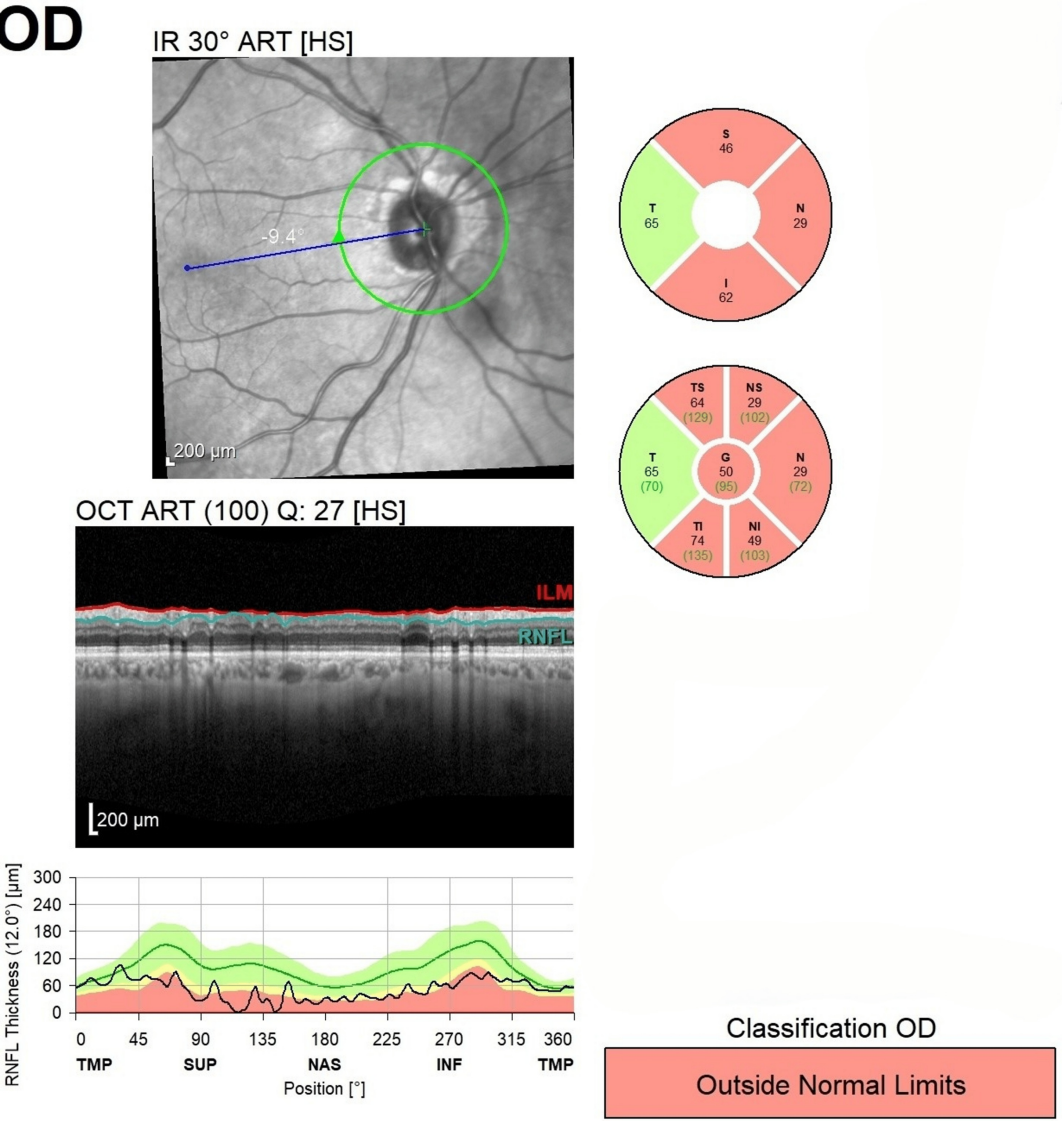
Optical Coherence Tomography (OCT) of the Right Eye (OD) Manifesting Decreased Retinal Nerve Fiber Layer (RNFL) Thickness in Superior, Inferior, and Nasal Quadrants ART, automatic real time; HS, high sensitivity; S, superior; N, nasal; T, temporal; I, inferior; G, global; TMP, temporal-macular profile

Optical coherence tomography (OCT) indicated nerve fiber layer thinning and retinal nerve fiber layer reduction on the right side in the superior, inferior, and nasal quadrants (Figures 1, 2). Brain magnetic resonance imaging (MRI) and orbits were conducted and did not show demyelination, infarct, or mass lesion. These laboratory test results showed a below normal status of folate in the serum, and a normal level of vitamin B12 was found. There were no variations in inflammatory markers, including hemoglobin, ESR, and HbA1c, and autoimmune markers, such as autoimmune serologies (antinuclear antibodies {ANA} and antineutrophil cytoplasmic antibodies {ANCA}), were negative (Table 1).

**Table 1:** Patient Laboratory Investigations With Corresponding Reference Values WCC, white cell count; Hb, hemoglobin

Test	Result	Reference Value (Unit)
CRP	4	<10 mg/L
ESR	2	0-35 mm/hour
Vitamin B12	930	211-911 pg/mL
Folate	5.1	>5.4 ng/mL
WCC	5.6	4.00-11.00×10^9^/L
Hb	145	120-150 g/L
HbA1c	36	<48 mmol/mol

These findings individually and collectively justified the diagnosis of methotrexate-induced optic neuropathy. Methotrexate was stopped, and replacement treatment with intramuscular vitamin B12 and oral folate was administered. The aforementioned case was subsequently referred to rheumatology for another form of immunomodulatory management; the patient is currently on sulfasalazine, and rheumatology is considering the commencement of an anti-tumor necrosis factor (TNF) agent for further disease control. Although methotrexate-induced optic neuropathy typically presents bilaterally, unilateral optic atrophy may occur due to the asymmetric vulnerability of the optic nerves, subclinical pre-existing damage, or localized vascular compromise. In this case, the right eye was affected first, and long-term follow-up is warranted to monitor potential involvement of the left eye. At a six-week follow-up, vision was still not normal; corrected visual acuity in the right eye was 6/18 and in the left eye 6/12, but it was no longer deteriorating further. A limitation of this is the short duration of follow-up. Long-term follow-up is planned to assess the progression or recovery of visual function and optic nerve changes. It also emphasizes that early detection and a timely approach toward possible neuro-ophthalmic complications of methotrexate should be a consideration.

## Discussion

This case highlights a rare but clinically significant adverse effect of methotrexate (MTX)-induced optic neuropathy. Fewer than 10 cases have been reported in the literature, most involving elderly patients receiving long-term low-dose MTX with concurrent folate or vitamin B12 deficiency [4-7]. In many of these reports, the discontinuation of MTX and appropriate vitamin replacement resulted in partial or complete visual recovery, indicating that MTX-induced optic neuropathy may be reversible if detected early [12,13].

MTX-induced neurotoxicity results from multiple interconnected mechanisms. MTX inhibits dihydrofolate reductase, reducing tetrahydrofolate availability and impairing thymidylate and purine synthesis, which disrupts DNA repair, myelin stability, and neuronal methylation pathways [3,4]. The optic nerve's high metabolic demand makes it vulnerable to mitochondrial dysfunction; recent studies demonstrate reduced cytochrome c oxidase activity, increased reactive oxygen species generation, and impaired mitochondrial oxidative phosphorylation in toxic and nutritional optic neuropathies [14,15]. Elevated homocysteine, common in folate and B12 deficiency, may further exacerbate neurotoxicity through oxidative stress and microvascular endothelial injury, compounding the effects of MTX [5,9].

Several case reports support these mechanistic pathways. Wang and Peng described bilateral optic neuropathy in a patient receiving 10 mg/week MTX with low folate and B12, showing recovery after discontinuation [4]. Wyse et al. [5] and Clare et al. [6] reported similar findings. Li et al. also reported that patients with reduced folate stores and those receiving concomitant proton pump inhibitors were at significantly higher risk of MTX-related toxicities, suggesting a similar susceptibility mechanism for MTX-induced visual complications [16]. A systematic review by Alruwaili et al. highlighted that isolated folate and B12 deficiencies can independently produce optic neuropathy by disrupting mitochondrial function and myelination, with favorable outcomes when treated early [10]. Grzybowski et al. highlighted that folate-responsive optic neuropathy can closely resemble other toxic optic neuropathies, emphasizing that timely recognition and treatment may lead to significant visual recovery [11].

In the present case, the patient developed subacute unilateral vision loss with normal MRI and inflammatory markers. OCT revealed retinal nerve fiber layer thinning, and laboratory evaluation confirmed folate deficiency with borderline B12 levels. Vision stabilized after MTX withdrawal and supplementation, though mild optic nerve pallor persisted, paralleling the partial recovery described in previous reports [8,12].

Although routine ophthalmological screening is not recommended for patients receiving methotrexate, certain red flags should prompt urgent evaluation. These include advanced age, the long-term use of MTX, low folate or vitamin B12 levels, and conditions that impair nutrient absorption, such as celiac disease or prior gastric surgery. Additional risk factors include chronic alcohol consumption, pre-existing optic nerve disease, and the emergence of new visual symptoms. Patients presenting with any of these features should undergo prompt ophthalmological assessment to prevent potentially irreversible visual loss.

Given the potential for irreversible optic atrophy, clinicians must maintain a high index of suspicion when visual complaints occur in patients receiving MTX. The early recognition of deficiency states, the timely discontinuation of the drug, and the prompt initiation of vitamin repletion can significantly improve outcomes. This case reinforces the need for heightened vigilance, particularly in older patients undergoing prolonged low-dose MTX therapy.

## Conclusions

Methotrexate-induced optic neuropathy, though rare, should be considered an important differential diagnosis in patients receiving long-term methotrexate therapy who present with new visual symptoms. Elderly individuals and those with pre-existing ophthalmic disease, such as glaucoma, diabetic or hypertensive retinopathy, age-related macular degeneration, or prior optic nerve pathology, as well as those with folate or vitamin B12 deficiency, appear to be at increased risk of methotrexate-related neurotoxicity. The early recognition and prompt cessation of methotrexate, alongside appropriate nutritional supplementation, may help prevent irreversible visual loss. Clinicians are advised to maintain a high index of suspicion and to seek ophthalmological evaluation in any methotrexate-treated patient reporting visual disturbances. In this case, at the six-week follow-up, right-eye vision remained stable without further deterioration, and left-eye visual acuity was normal. The patient was receiving vitamin B12 and folic acid supplementation and had been transitioned to an alternative immunomodulatory agent for the management of psoriatic arthritis.
